# The Effects of Interphase and Interpulse Delays and Pulse Widths on Induced Muscle Contractions, Pain and Therapeutic Efficacy in Electroporation-Based Therapies

**DOI:** 10.3390/jcdd10120490

**Published:** 2023-12-07

**Authors:** Aleksandra Cvetkoska, Alenka Maček-Lebar, Tamara Polajžer, Matej Reberšek, Weston Upchurch, Paul A. Iaizzo, Daniel C. Sigg, Damijan Miklavčič

**Affiliations:** 1 Faculty of Electrical Engineering, University of Ljubljana, 1000 Ljubljana, Slovenia; aleksandra.cvetkoska@fe.uni-lj.si (A.C.); alenka.macek-lebar@fe.uni-lj.si (A.M.-L.); tamara.polajzer@fe.uni-lj.si (T.P.); matej.rebersek@fe.uni-lj.si (M.R.); 2 Visible Heart® Laboratories, Department of Surgery and the Institute for Engineering in Medicine, University of Minnesota, Minneapolis, MN 55455, USA; wupchurc@umn.edu (W.U.); iaizz001@umn.edu (P.A.I.); 3 Cardiac Ablation Solutions, Medtronic, Inc., Minneapolis, MN 55432, USA; daniel.c.sigg@medtronic.com

**Keywords:** pulsed-field ablation, atrial fibrillation, electroporation, pulse waveform, pulse parameters, nerve and muscle stimulation

## Abstract

Electroporation is used in medicine for drug and gene delivery, and as a nonthermal ablation method in tumor treatment and cardiac ablation. Electroporation involves delivering high-voltage electric pulses to target tissue; however, this can cause effects beyond the intended target tissue like nerve stimulation, muscle contractions and pain, requiring use of sedatives or anesthetics. It was previously shown that adjusting pulse parameters may mitigate some of these effects, but not how these adjustments would affect electroporation’s efficacy. We investigated the effect of varying pulse parameters such as interphase and interpulse delay while keeping the duration and number of pulses constant on nerve stimulation, muscle contraction and assessing pain and electroporation efficacy, conducting experiments on human volunteers, tissue samples and cell lines in vitro. Our results show that using specific pulse parameters, particularly short high-frequency biphasic pulses with short interphase and long interpulse delays, reduces muscle contractions and pain sensations in healthy individuals. Higher stimulation thresholds were also observed in experiments on isolated swine phrenic nerves and human esophagus tissues. However, changes in the interphase and interpulse delays did not affect the cell permeability and survival, suggesting that modifying the pulse parameters could minimize adverse effects while preserving therapeutic goals in electroporation.

## 1. Introduction

Electroporation, a technique widely employed for various biomedical applications, entails the application of high-voltage electric pulses to tissues, allowing for the temporary permeabilization of cell membranes, increasing the potential for either drug delivery or gene therapy [1,2,3,4,5,6]. Furthermore, pulsed-field ablation (PFA) is a specific application of irreversible electroporation, rapidly substituting thermal energies in cardiac ablation procedures [7,8,9]. These ablative procedures are used with the intent to permanently disrupt abnormal electrical pathways and thus restore a normal heart rhythm. One of the considered primary advantages of PFA is its ability to create precise and controlled lesions, allowing for the accurate targeting of specific areas while minimizing damage to surrounding healthy tissue, or collateral damage [10,11,12,13,14,15,16]. Additionally, PFA clinical procedures require less time in comparison to the currently used techniques for cardiac ablation [17].

Although PFA is generally considered safe, it is important to acknowledge the potential occurrence of adverse effects and complications related to any such procedure [18,19]. During a PFA treatment, the electrical pulses are delivered to the target treatment site, e.g., the pulmonary veins of the left atrium. While the region subjected to irreversible electroporation (IRE) is the nearest to the ablation electrodes, the electric fields generated during PFA treatment can extend significantly beyond the zone of therapeutic IRE, leading to the unwanted stimulation of neuromuscular structures. For example, when using 100 µs pulses, nerves are excited with an electric field strength that is two orders of magnitude lower than the field strength required for IRE [20,21,22], which means that pulsed fields can excite certain tissues, including nerves and both smooth and striated muscles (e.g., skeletal muscles), which can lead to patient discomfort and/or involuntary muscle movements. Some pulsed fields are also likely to directly stimulate nociceptors and/or pain nerve fibers (e.g., A-delta and/or C fibers) leading to the corresponding sensation of pain [23]. The stimulation of vagal structures can result in bradycardia and/or cough reflexes. These adverse effects, including muscle contractions, pain, discomfort, bradycardia, and/or coughing, can have procedural implications [24]. Therefore, the administration of sedatives and/or anesthetics during cardiac ablation is required. Therapeutic strategies to mitigate these unwanted adverse effects involve changing pulse parameters/waveforms or the modification of anesthetic protocols (e.g., the use of paralytics). It should be noted, however, that in many geographies (e.g., Europe, Japan), it is undesirable to conduct ablation procedures, such as pulmonary vein isolations, under general anesthesia. 

Early attempts to reduce muscle contractions and pain during high-voltage pulse delivery for drug delivery were directed at increasing pulse repetition rates while maintaining the pulse number and pulse width. Initial studies showed limited success—causing more severe contractions but a lower number of contractions [25,26]—but also slightly reduced the electroporation’s efficacy. Shorter pulses (in the nanosecond range) were suggested to alleviate nerve and muscle contractions while maintaining therapeutic efficacy [21,27,28], but the technology to deliver such pulses is not readily available [29,30]. Most recently, high-frequency biphasic pulses were suggested for the irreversible ablation of soft tissues, which apparently alleviate muscle contractions; i.e., using short pulses in the range of 1 to 10 μs pulse widths [20,31,32,33,34,35,36]. However, these studies have only observed reduced contractions when the interphase (delay between the positive and negative phase) and interpulse (delay between the pulses) delays were equal. Furthermore, the therapeutic efficacies of these pulses were shown to be greatly reduced in terms of membrane permeabilization and/or induced cell death. This reduced efficacy requires the use of electric pulses with increased amplitudes, even up to 2–3 times higher, as observed in in vitro and in vivo studies [32,37] when compared to longer (e.g., 100 μs) pulses of equal total on-time. 

The exploration of modifying interphase and interpulse delays for reducing excitation in these protocols has not been investigated until recently, when it was proposed in a theoretical study [22]. Extending interpulse delays while minimizing the interphase delays was suggested to achieve a reduction in nerve excitation. Of interest, in a first human study [38] by our group, it was demonstrated that, in addition to pulse widths and pulse repetition rates, interphase and interpulse delays were important pulse parameters. We showed that shortening the interphase delays (1–2 µs) and extending the interpulse delays (above 100 µs) can potentially lower the associated muscle contractions during the treatments, which means that the thresholds for stimulation increased (this is in agreement with the theoretical/numerical study by Aycock et al. [22]). These observations show that the interplay between pulse width, interphase delay and interpulse delays is complex, and that the modification of these parameters can reduce nerve excitation. However, it is not clear how these parameters can affect electroporation’s efficacy. Namely, short pulses and short interpulse delays were reported to be less efficient—the phenomenon was named the cancelation effect [39].

In our study, we examined the muscle contractions caused by biphasic pulses using a wide range of different pulse wave parameters (pulse width, interphase and interpulse delay). We also assessed the pain sensation in healthy volunteers that occurs during muscle stimulation. Using the subset of pulse parameters, we also performed ex vivo stimulation on porcine phrenic nerves and human esophagus tissues, as well as in vitro experiments to determine cell membrane permeabilization and cell survival in different cell lines. 

## 2. Materials and Methods

### 2.1. Muscle Stimulation and Pain Study

#### 2.1.1. Study Design

Participants: This study was approved by the National Medical Ethics Committee of Slovenia (Doc. no. 0120-558-2021) and was conducted following the Declaration of Helsinki, the Convention on Human Rights and Biomedicine (ETS No.164), and the Slovenian Code of Medical Ethics. Sixteen healthy individuals (8 females and 8 males) volunteered to participate in this study in the age range from 23 to 59 years. Informed consent was obtained from all volunteers before the start of the measurements. All of them were given the opportunity to withdraw from the study at any time.

**Figure 1:**
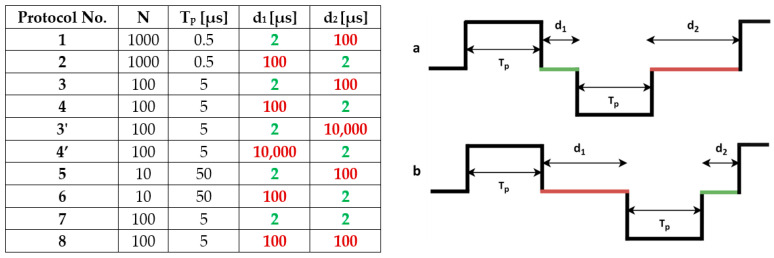
Values of the pulse parameters for the biphasic pulse protocols included in the study. All protocols have equal total on-time, i.e., N × 2T_p_ = 1 ms. T_p_—pulse width (equal for positive and negative phase); N—number of pulses; d_1_—interphase delay; d_2_—interpulse delay. (**a**) Scenario one: short d_1_ and long d_2_ (protocols 1, 3, 3′ and 5); (**b**) scenario two: long d_1_ and short d_2_ (protocols 2, 4, 4′ and 6). Additionally, protocols 7 and 8 (with equal d_1_ and d_2_) were added for comparison and tested in the ex vivo study.

Protocols: Based on a recent theoretical/numerical study [22] and our previous human study [38], we wanted to further investigate whether minimizing the interphase delay (d_1_) while extending the interpulse delay (d_2_) reduces the muscle contraction responses and pain for different pulse widths. Therefore, we selected 3 pulse widths (0.5 μs, 5 μs and 50 μs). For each pulse width, we tested two scenarios: one with a short d_1_ and long d_2_ (protocols 1, 3, 3′ and 5) and one with a short d_2_ and long d_1_ (protocols 2, 4, 4′ and 6), as shown in Figure 1.

#### 2.1.2. Test Procedure 

**Figure 2:**
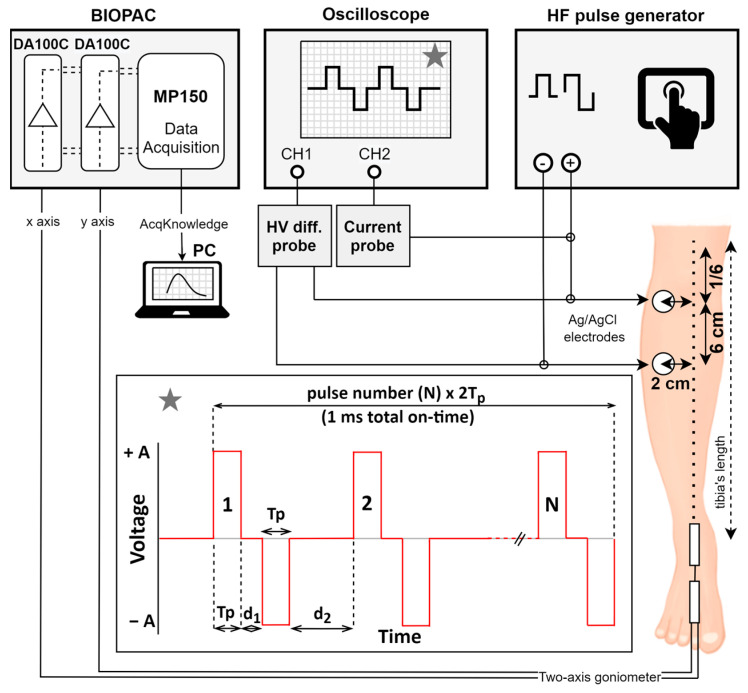
Experimental setup and electrode/goniometer placement. The stimulation pulses were delivered via electrodes connected to the high-frequency (HF) pulse generator. The electrodes (marked with circles) were placed on the right leg: the upper electrode was placed on 1/6th of the tibia’s length; the lower electrode was placed 6 cm lower. Both electrodes were placed 2 cm right lateral to the bone (left in the figure). The output pulses were monitored on an oscilloscope using high-voltage (HV) differential and current probe. Asterisk: applied pulses—biphasic pulses with 1 ms total on-time. Tp—pulse width (equal for positive and negative phase), d_1_—interphase delay, d_2_—interpulse delay, N—number of pulses. The response from the ankle (muscle contraction response) was acquired with twin-axis goniometer connected to the Biopac unit (x-axis (channel) determining foot dorsiflexion/plantarflexion, y-axis (channel) determining foot abduction/adduction). The data were analyzed on a personal computer (PC) using the AcqKnowledge software 4.0 . DA100C—amplifier, MP150—data acquisition system. Adapted from [38].

The measurements were conducted in the same way as previously described [38]. Briefly, the complete experimental set up is shown in Figure 2. The volunteers were seated with their legs relaxed. The study duration was approximately one hour for each individual. No anesthetics or nerve blockers were used. For the delivery of electrical pulses, a prototype high-frequency (HF) pulse generator was used (L-POR V 0.1, mPOR, Ljubljana, Slovenia), with an internal alternation for safe human use (lower capacitance of the capacitors and fast discharging), along with two self-adhesive Ag/AgCl electrodes (3 M™ Red Dot™, 3M, Saint Paul, MN, USA) placed on the skin above the middle of the tibialis anterior muscle. The lowest amplitude limit of the pulse generator was 60 V; the highest amplitude limit was 1000 V. To assess for muscle contractions, the angle of ankle dorsiflexion was measured with a twin axis goniometer TSD120B (Biopac Systems, Inc., Goleta, CA, USA) attached to the given patient’s ankle. Two planes of angular movements were simultaneously measured (x-axis: foot dorsiflexion/plantarflexion; y-axis: abduction/adduction). Each channel (x, y) of the goniometer was connected to a single DA100C amplifier as part of Biopac’s MP150 data acquisition system.

The measurements were only performed for 3 randomly (https://www.calculatorsoup.com/, accessed on 15 November 2022, Ashland, MA, USA) chosen protocols (Figure 1) on each volunteer. For each protocol, the minimal muscle response (stimulus intensity, i.e., pulse amplitude) that would cause ankle dorsiflexion of about 3.6° up to 4° was first determined. The amplitude was then increased (from low to high) in small increments until (1) maximal muscle response (plateau of the muscle or strong muscle response also on the second channel—foot abduction/adduction); (2) the amplitude that the device was able to deliver (1000 V) was reached; or (3) the volunteer chose to withdraw. By gradually increasing the amplitude, we made sure that the pain was considered bearable for each subject. Note that, after each amplitude increase, the volunteers had the right to withdraw from the study. 

A short-form McGill Pain Questionnaire (PQ) was completed by each volunteer for all protocols delivered, immediately after each pulse delivery. First, the pain rating index (PRI) was employed, which was used to determine the pain descriptors (1–8) rated on an intensity scale (0–3). Next, two separate (0–10 cm) horizontal visual analog scales (VASs) were provided which were used to assess pain intensity and unpleasantness. At the end, each volunteer was given a paper with the third section of the PQ to evaluate whether there were some visible signs of injury or pain 6 h after the pulse delivery. They were requested to send these answers via e-mail.

#### 2.1.3. Data Analyses

Muscle responses: The responses (obtained with the goniometer as .matlab files) were filtered and analyzed in MATLAB vR2018a (MathWorks Inc., Natick, MA, USA) to determine the angle of ankle dorsiflexion for each protocol and amplitude delivered (for each individual separately). Each protocol was repeated 8 times. The mean value for the angle was then calculated for each protocol and amplitude (only for the amplitudes that were tested per a given individual). 

Pain Questionnaires: The total pain index for each subject was calculated from the pain questionnaire for each protocol and amplitude delivered. The total pain index was calculated as a sum of the pain rating index (PRI) and both visual analog scales (VASs). The PRI was derived from the sum of the intensity rank values of the words chosen by each individual for defined sensory and affective descriptors (8 pain descriptors, scale: 0–3). The VAS analyses consisted of measuring the distance in centimeters with a ruler between the start of the line on the left side and the mark made by the individual (scale: 0–10). The total pain index was normalized per individual and protocol (values between 0 and 1). The mean value for the total pain index was then calculated for each protocol and amplitude (only for the amplitudes that were tested per each individual). 

Statistical analyses: Comparisons of the mean values were made between each set of protocols (1 and 2, 3 and 4, and 5 and 6) for each amplitude employed. A *t*-test with a level of significance set to 0.05 was performed in SigmaPlot 11.0 (Systat Software Inc., San Jose, CA, USA) within each set of protocols for both the muscle response and pain index. For some sets where normal distribution was not obtained, a Mann–Whitney rank sum test was performed.

### 2.2. Stimulation of Isolated Nerves and Muscles

Experiments on isolated nerves and muscles were performed within the Visible Heart^®^ Laboratories (University of Minnesota, Minneapolis, MN, USA). For this study, swine phrenic nerves and human esophagus tissues were isolated and dissected at room temperature within an oxygenated KREBS buffer; the mucosa–submucosa layers of the esophagus were removed, and transverse strips of the muscularis propria were prepared. For the isolated nerve experiments, the phrenic nerves were temporarily taken out from the 37 °C oxygenated KREBS buffer and then placed within the nerve recording chamber (MLT016, ADInstruments, Colorado Springs, CO, USA). To measure the elicited compound nerve action potentials (CNAPs), a given phrenic nerve was stimulated at threshold voltages employing different high-frequency biphasic pulse protocols; the phrenic nerves were stimulated at one end with the L-POR electroporator (mPOR, Slovenia). The CNAPs of a given phrenic nerve were measured 2 cm from the stimulus using a differential DAQ system. To determine the threshold voltage, the amplitude of the employed high-frequency biphasic pulse protocol was slowly increased until the elicited CNAP was reproducible: >200 µV in amplitude. Between each experiment, the phrenic nerve was placed back into the 37 °C oxygenated KREBS buffer recovery chamber. For the isolated muscle experiment, carefully prepared strips of dissected esophagus, 2–3 mm in diameter and more than 2 cm long, were placed in tissue baths [40]. Two high-frequency biphasic pulse protocols were applied to the electrodes in the tissue baths and the peak-to-peak force was measured using the DAQ system. Three repetitions were performed for each protocol and the standard deviations were calculated.

### 2.3. Experiments on Cell Permeability and Cell Survival

#### 2.3.1. Cell Lines

Cellular experiments were performed utilizing three cell lines with different tissue and species origins: Chinese hamster ovary (CHO) (cat #85051005), mouse melanoma cells (B16F1) (cat #92101023) and rat heart myoblast (H9c2) (cat #88092904). All cell lines were obtained from the European Collection of Authenticated Cell Cultures. The CHO cells were grown in HAM N6658, B16F1 v DMEM D5671 and H9c2 in DMEM D6546 (all from Sigma Aldrich, Darmstadt, Germany). All media were supplemented with 10% fetal bovine serum (Sigma Aldrich, ZDA), L-glutamine (StemCell, Vancouver, Canada) and antibiotics—penicillin/streptomycin (PAA) and gentamycin (Sigma Aldrich, ZDA). The cells were sub-cultured every 3–4 days and incubated at 37 °C in a humidified atmosphere with a 5% (CHO and B16F1) or 10% (h9c2) CO_2_ incubator. The cells were detached with a trypsin solution (10x trypsin-EDTA (PAA, Leonding, Austria) 1:9 diluted in Hank’s basal salt solution (StemCell, Canada), which was inactivated after 2–3 min through the addition of the fresh growth medium. The cells were centrifuged for 5 min at 180× *g* and 22 °C, after which the supernatant was removed and replaced with an appropriate fresh growth medium to a cell density of 1 × 10^6^ cells/mL. A total of 150 mL of the cell suspension was then transferred to a cuvette and therapeutic pulses were applied. 

#### 2.3.2. Pulse Deliveries

For the delivery of electrical pulses, a laboratory prototype high-frequency (HF) pulse generator was used (L-POR V 0.1, mPOR, Ljubljana, Slovenia). This generator has the capacity to produce pulse amplitudes reaching 1700 V, achieved through an internal alternation that involved the use of capacitors different from those utilized in the human study. The same pulse protocols as described above were used (forming one burst) with various electric field strengths. Furthermore, the burst numbers were also increased up to 10 bursts, delivered with burst repetition frequencies of 250 mHz, i.e., one burst delivered every 4 s. The delivered pulses were monitored using a high-voltage differential probe, HVD3605A (Teledyne LeCroy, Chestnut Ridge, NY, USA); a current probe, CP031 (Teledyne LeCroy, New York, NY, USA); and a HDO6000 High-Definition oscilloscope (Teledyne LeCroy, New York, NY, USA).

#### 2.3.3. Cell Membrane Permeability 

A total of 150 µL of the cell suspension was transferred into aluminum cuvettes with a 2 mm distance (VWR International, Radnor, PA, USA). Prior to pulse application, propidium iodide (PI, Life Technologies, Carlsbad, CA, USA) was added to the cells to a final concentration of 100 µg/mL. After pulse application, the cells were incubated for three minutes at room temperature and then analyzed using a flow cytometer (Attune NxT; Life Technologies, Carlsbad, CA, USA) using a 488 nm blue laser and a 574/26 nm band-pass filter; 10,000 events were collected and analyzed. Fluorescence intensity histograms were used to determine the percentage of PI-permeabilized cells. Gating was set according to a sham control (0 V). Each experimental protocol was repeated three times.

#### 2.3.4. Cell Survival

A total of 150 µL of the cell suspension was transferred into aluminum cuvettes with a 2 mm distance and pulses were delivered. Then, cells were diluted in the appropriate growth media and 2 × 10^4^ cells per well were seeded in 96-well plate (TPP, Trasadingen, Switzerland) and then incubated at 37 °C and humidified in a 5% CO_2_ atmosphere for 24 h. After the incubation period, 20 µL of an MTS tetrazolium compound (CellTiter 96 AQueous One Solution Cell Proliferation Assay, Promega, Sunnyvale, CA, USA) was added to the cells and incubated for an additional 2 h. Afterwards, the absorbance of the reduced MTS tetrazolium compound was measured with a spectrofluorometer (Tecan Infinite M200, Tecan, Grödig, Austria) at 490 nm. The percentage of viable cells was obtained via the subtraction of blanks and the normalization of sample absorbance to the absorbance of the sham control (0 V). Each measurement was made in three technical repetitions and each experimental data point was repeated three times.

## 3. Results

### 3.1. Muscle Stimulation and Pain Scores

**Figure 3:**
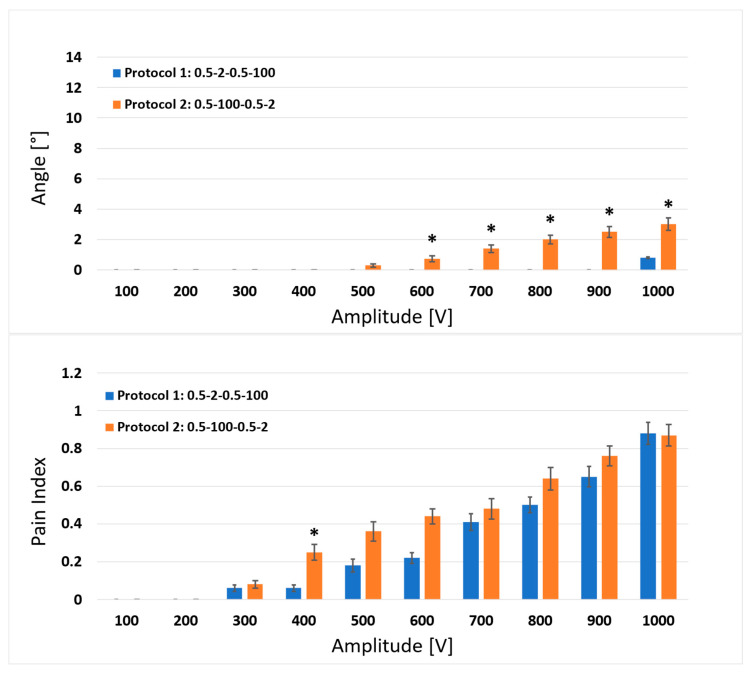
Comparison of the results between protocol 1 (0.5-2-0.5-100 µs) and protocol 2 (0.5-100-0.5-2 µs) for the muscle response (upper graph) and pain index (lower graph). Each bar represents one pulse protocol (T_p_-d_1_-T_p_-d_2_) for the stated amplitude on the x-axis. The results are shown as the mean value (bar’s height) ± standard error (black vertical bars). The asterisks (*) show statistically significant differences between the pulse protocols at the stated amplitude (*p* < 0.05).

**Figure 4:**
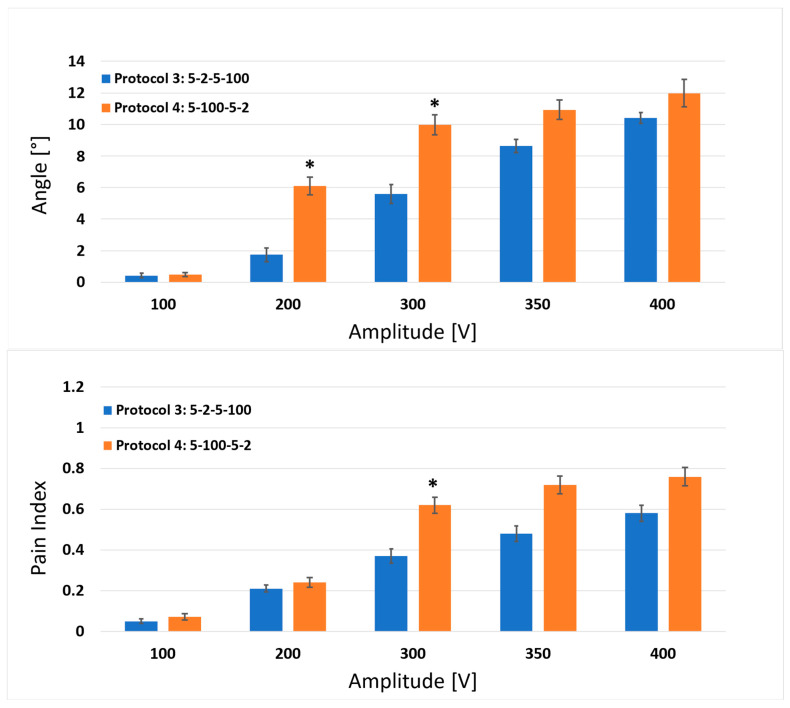
Comparison of the results between protocol 3 (5-2-5-100 µs) and protocol 4 (5-100-5-2 µs) for the muscle response (upper graph) and pain index (lower graph). Each bar represents one pulse protocol (Tp-d_1_-Tp-d_2_) for the stated amplitude on the x-axis. The results are shown as the mean value (bar’s height) ± standard error (black vertical bars). The asterisks (*) show statistically significant differences between the pulse protocols at the stated amplitude (*p* < 0.05).

**Figure 5:**
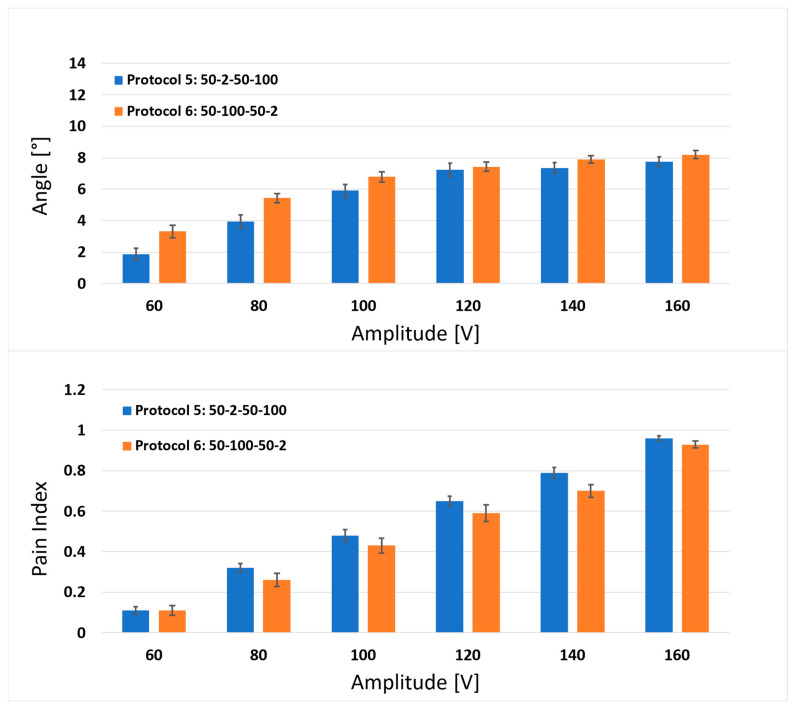
Comparison of the results between protocol 5 (50-2-50-100 µs) and protocol 6 (50-100-50-2 µs) for the muscle response (upper graph) and pain index (lower graph). Each bar represents one pulse protocol (T_p_-d_1_-T_p_-d_2_) for the stated amplitude on the x-axis. The results are shown as the mean value (bar’s height) ± standard error (black vertical bars).

In Figure 3, Figure 4 and Figure 5, we present the results obtained for the in vivo human muscle stimulation and pain study for the six high-frequency biphasic pulse protocols tested (Figure 1). The mean results with the corresponding standard errors for muscle responses (upper figure) and pain indexes (lower figure) are shown for each pair of protocols (comparisons 1–2, 3–4 and 5–6). Statistically significant differences were observed between pulse protocols 1 and 2 (Figure 3) for the delivered amplitudes of 600, 700, 800, 900 and 1000 V for the muscle response, and only for the amplitude of 400 V for the pain index. 

For protocols 3 and 4 (Figure 4), statistically significant differences were observed for amplitudes of 200 and 300 V for the muscle response, and only for the amplitude of 300 V for the pain index. For protocols 5 and 6 (Figure 5), no statistically significant differences were observed between the protocols. In summary, simultaneously changing the d_1_ from 2 to 100 µs and the d_2_ from 100 to 2 µs induced more pronounced muscle responses (greater angle, stronger muscle contraction) for each observed set of protocols and amplitudes, whereas for the pain index, the same trend was observed only for the comparisons between protocols 1–2 and 3–4. The comparisons of the pain indexes for protocols 5 and 6 showed that the pain index was slightly lower (although not significantly) when interchanging the d_1_ from 2 to 100 µs and the d_2_ from 100 to 2 µs.

### 3.2. Stimulation of Isolated Phrenic Nerves and Esophageal Muscles

**Figure 6:**
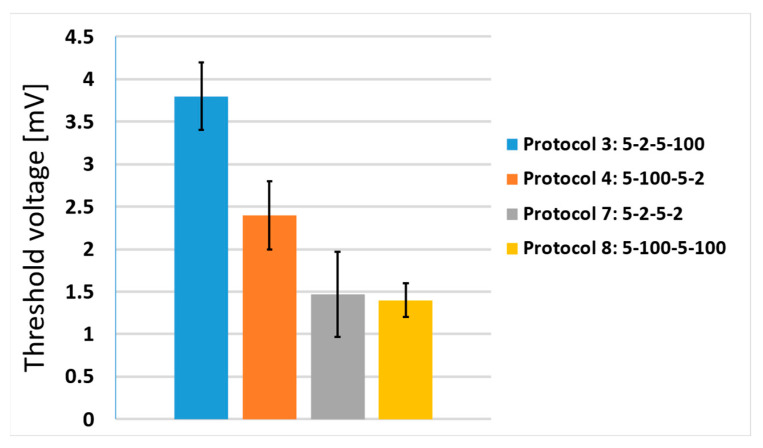
Threshold voltage of phrenic nerve stimulations at biphasic protocols with different interphase delays (d_1_) and interpulse delays (d_2_). All protocols had the same number of pulses (100) and pulse width (5 µs). Each bar represents one pulse protocol (blue—protocol 3, orange—protocol 4, gray—protocol 7, yellow—protocol 8). The results are shown as the mean value of three repetitions (bar’s height) ± standard deviation (black vertical bars).

In Figure 6, we present the results of the phrenic nerve stimulation study. Four different protocols were tested in this study: protocols 3, 4, 7 and 8 (see Figure 1). Note that the latter two protocols were not tested in the clinical study described in Section 2.1 and Section 3.1. All protocols had the same number of pulses (100) and pulse width (5 µs); only the interphase delay (d_1_) and interpulse delay (d_2_) were different. It can be observed that protocol 3 (5-2-5-100 µs) resulted in the highest threshold voltage compared to the other tested protocols. Interestingly, protocols 7 and 8 had almost the same threshold voltage, both being lower than the threshold voltage for protocol 4.

**Figure 7:**
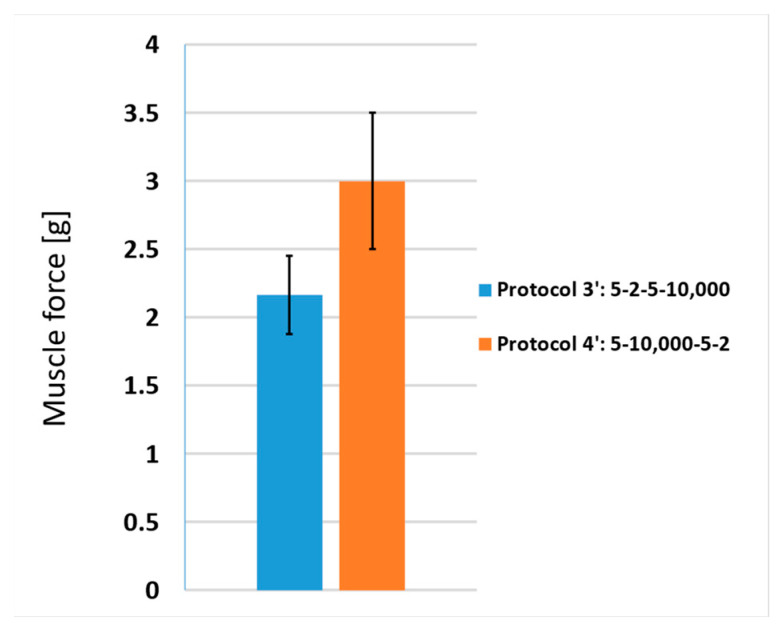
Muscle force during stimulations of esophagus muscle strips at biphasic pulse protocols with different interphase delays (d_1_) and interpulse delays (d_2_). Both protocols had the same number of pulses (100) and pulse width (5 µs). Each bar represents one pulse protocol (blue—protocol 3′, orange—protocol 4′). The results are shown as the mean value of three repetitions (bar’s height) ± standard deviation (black vertical bars).

In Figure 7, we present the results of the esophagus stimulation studies; two different protocols were tested. Both protocols employed the same number of pulses (100) and pulse widths (5 µs), while the interphase delay (d_1_) and interpulse delay (d_2_) were alternated. Due to the low impedance of the muscle baths (6–8 Ω), the protocols were adjusted to have longer delays (10,000 µs instead of 100 µs) in order to refill the capacitors as required. Thus, protocols 3 and 4 were modified into protocols 3′ (5-2-5-10,000 µs) and 4′ (5-10,000-5-2 µs). It can be observed that protocol 3′ elicited lower muscle forces compared to protocol 4′, which is in accordance with the results presented in Figure 4.

### 3.3. Therapeutic Efficacy: Experiments on Cell Membrane Permeability and Cell Survival

**Figure 8:**
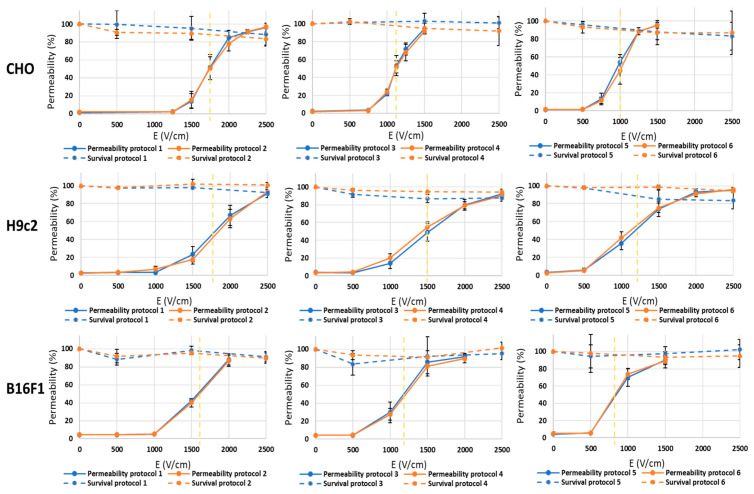
Cell membrane permeability and cell survival for protocols 1–6 for three different cell lines: CHO (first row), H9c2 (second row) and B16F1 (third row). The results are shown as the mean value of three repetitions (each point) ± standard deviation (black solid vertical bars). The yellow dashed vertical lines present the threshold values (determined at 50% permeability).

The in vitro effects of the parameters on the induced efficiency between pulses with a short d_1_ and long d_2_ and pulses with a short d_2_ and long d_1_ of different pulse widths were investigated. The protocols tested were identical to what is described in Figure 1 (protocols 1–6). When the pulses were delivered as one burst, no differences were observed between pulses with a short d_1_ and long d_2_ and pulses with a short d_2_ and long d_1_. This was a consistent finding in all three cell lines (Figure 8). The only difference between the pulse protocols was in the permeabilization thresholds (i.e., 50% permeabilization values, as indicated with the vertical dashed yellow lines in Figure 8). Shorter pulse widths resulted in higher thresholds than longer pulse widths, regardless of the cell line. This is in agreement with previous published studies, as shorter pulse widths require higher pulse amplitudes (or more bursts) to obtain a comparable effect as with longer pulse widths [32,41]. 

**Figure 9:**
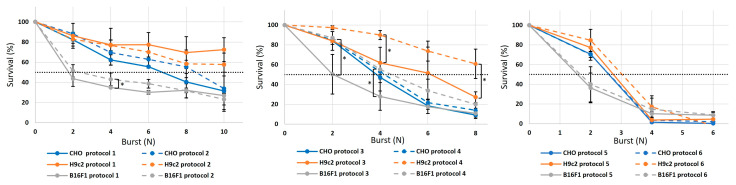
Increasing the burst number for protocols 1–6 for three different cell lines (CHO, H9c2 and B16F1): protocol 1–2 (first left graph), protocols 3–4 (middle graph) and protocols 5–6 (last right graph). The results are shown as the mean value of three repetitions (each point) ± standard deviation (black vertical bars). The asterisks (*) show statistically significant differences between the pulse protocols at the stated number of bursts (*p* < 0.05). The black dashed horizontal lines determine 50% cell survival.

While one burst of pulses was sufficient to achieve increased membrane permeability, it had no effect on survival. Therefore, we increased the number of bursts while keeping a fixed voltage of 500 V (2500 V/cm). In all three cell lines, increasing the number of pulses/bursts resulted in increased cell death (Figure 9). However, not all pulse protocols had the same efficacy on cell death. A comparison of the therapeutic pulses with a short d_1_ and long d_2_ and those pulses with a short d_2_ and long d_1_ shows that they appear slightly different in their effects within the different cell lines. Yet, the comparisons between protocols 1 and 2 elicited no difference in cell death efficacy in CHO and H9c2, while in B16F1, a difference was observed only when four bursts were delivered. A comparison between protocols 3 and 4 resulted in some differences, while the majority of experimental points were the same. No differences were observed in the CHO cell line; in H9c2, the differences were observed at four and eight bursts, and in B16F1 at two and four bursts. The observed differences were in the value range of 20–30%. Note that the standard deviation in some of these results was in the same 20–30% range. No differences were observed between the responses to protocols 5 and 6, where the increase to four bursts led to 90% cell death, regardless of the cell line. Furthermore, we observed no significant differences in cell survival in different cell lines. Overall, when the delivered pulse widths were shorter, a higher number of bursts were needed to achieve 50% cell death/survival in spite of the equal total pulse duration. With protocols 5 and 6, we achieved less than 20% survival with four bursts of pulses; when protocols with shorter pulses were used, we again (as in the permeabilization assay) observed a reduced efficacy in terms of cell death. Interestingly the cell survival when exposed to these protocols with shorter pulses was even better in H9c2 than in CHO and B16F1, contrary to some other in vitro reports [42,43].

## 4. Discussion

Electroporation has become a valuable tool for applications like drug and gene delivery, tumor ablation and, most recently, cardiac ablation (pulsed-field ablation, PFA). However, an adverse effect of this technique is neuromuscular stimulation. Nerve stimulation can cause discomfort and muscle contractions and trigger the release of neurotransmitters, such as acetylcholine (ACh), by the parasympathetic nervous system within the heart [44]. This, in turn, decreases the heart rate and the rate of conduction, possibly accounting for post-PFA bradycardia [24]. Without the administration of prophylactic treatment, typically involving the injection of an anticholinergic drug (atropine), these adverse effects may not be acceptable for patients.

The primary aim of the human trial was to explore the impact of the varying therapeutic pulse parameters (such as pulse widths and interphase and interpulse delays) on induced muscle contractions, as well as evaluating the perception of pain during the stimulation with high-frequency biphasic pulse protocols. We then applied these identical pulse parameters within ex vivo stimulation experiments on isolated swine phrenic nerves and human esophagus tissues. Furthermore, we conducted in vitro cell experiments to determine the effects on membrane permeabilities and viabilities using various cell lines. With the combined data acquired from this presented study, we provide evidence to develop novel therapeutic protocol options aimed at mitigating or minimizing the unwanted adverse effects observed during the applications of PFA and potentially in other electroporation-based treatments.

Our results obtained in healthy individuals confirm that short high-frequency biphasic pulses with short interphase and long interpulse delays can reduce both associated muscle contractions (a higher threshold voltage is obtained) and pain sensations in comparison with delivered pulses with long interphase and short interpulse delays. The stimulation of nerves and muscles has been extensively investigated in the past, showing that short pulses and higher frequencies of alternating currents (up to 10 kHz) can increase sensory, motor and pain thresholds [45,46,47,48]. Thus, to minimize the stimulation of muscles and nerves during electroporation-based treatments, an increase in the pulse repetition frequency far above the frequency of tetanic contraction was suggested [25]. Moreover, alternating the current with a frequency of 300 kHz to 1 MHz is also used for surgical procedures (cutting, coagulating of biological tissue), as the threshold current for the excitation of nerves and muscles increases with frequency [49]. In our study, very short pulses (0.5 μs) with short interphase and long interpulse delays were associated with the lowest muscle contraction and pain scores, which is consistent with the results obtained in our previous human study [38]. The in vitro stimulation experiments on swine phrenic nerves and human esophagus tissues also revealed higher threshold voltages for the protocols with short interphase and long interpulse delays. Thus, a longer interphase delay may be less desirable than a longer interpulse delay when designing novel clinical electroporation waveforms. When the same protocols were tested in the cell permeability and survival experiments, no differences were observed between the protocols with short interphase and long interpulse delays and protocols with short interpulse and long interphase delays, in each of the three different cell lines. Overall, our results suggest that switching between short and long interphase and interpulse delays can influence the nerve stimulation thresholds but not the cell permeability and survival. Therefore, the overall results presented in this study suggest that the pulse wave parameters can be selected, modified and/or adjusted, such that adverse effects like pain, contraction and coughing may be reduced (i.e., minimized) while the intended therapeutic purposes are still achieved. However, it should be considered that longer delays between the pulses might also be required in order to minimize potential thermal effects [50,51]. 

High-voltage electric pulses used in electroporation-based treatments can inadvertently stimulate peripheral nerves, triggering action potentials that then propagate towards the muscles. This activation of motor neuron axons initiates muscle contractions, which can result in movements of the innervated muscles (locally or at distant sites). Peripheral nerve stimulation associated with functional electrical stimulation has long been known and studied. Here, relatively long pulses were used to initiate muscle function. The activation of nerves innervating the muscles is the most likely mechanism for muscle stimulation rather than direct muscle fiber stimulation [52]. Once motor neurons are activated, a cascade of events initiates muscle contraction. The excitation signal travels from the motor neurons to the neuromuscular junction, where acetylcholine is released. Acetylcholine then binds to receptors on muscle fibers, initiating intracellular processes involving calcium release, actin–myosin interactions and subsequent muscle contraction. These muscle contractions, when affecting the diaphragm or surrounding muscles in the chest area, can in turn induce coughing or hiccups. 

The cough reflex is a physiological reflex involving receptors in the upper airways, the vagal nerve (C-fibers and myelinated fibers), the glottis/epiglottis and expiratory muscles of the abdomen and thorax to clear the airways from the left superior pulmonary vein. There is close anatomical proximity between the LSPV (left superior pulmonary vein) and the LSMB (left stem main bronchus) which can allow its direct stimulation by electric pulses. The stimulation of the cough reflex has been reported in the literature and seems to be a plausible mechanism [53]. Interestingly, in other, i.e., thermal, ablation modalities, coughing is a sign of bronchial damage [54] or phrenic nerve injury [55]. Coughing has been observed during clinical pulsed-field ablation procedures [12,13,56]. Patient movement due to coughing can result in map shifts if electro-anatomical mapping systems are utilized, and therefore should be avoided. 

Unlike neuromuscular activation, pain is a complex subjective sensory phenomenon, involving nociceptors (specialized afferent neurons responsible for detecting potentially harmful stimuli), peripheral nerves, the spinal cord and the sensory cortex within the brain. The activation of these nociceptors triggers the transmission of pain signals from the peripheral nerves to the brain, resulting in the perception of pain even without focal injury. Also, the stimulation of the autonomic nervous system can enhance or inhibit organ activity. The stimulation of the sympathetic nervous system typically leads to the release of norepinephrine (NE) from postganglionic neurons within the organ, while the stimulation of the parasympathetic nervous system triggers the release of acetylcholine (ACh) from postganglionic neurons in the organ. In the case of the heart, the stimulation of the sympathetic nervous system will increase the heart rate, force of contraction and rate of conduction. Conversely, the stimulation of the parasympathetic nervous system leads to a reduction in heart rate and the rate of conduction [44].

**Figure 10:**
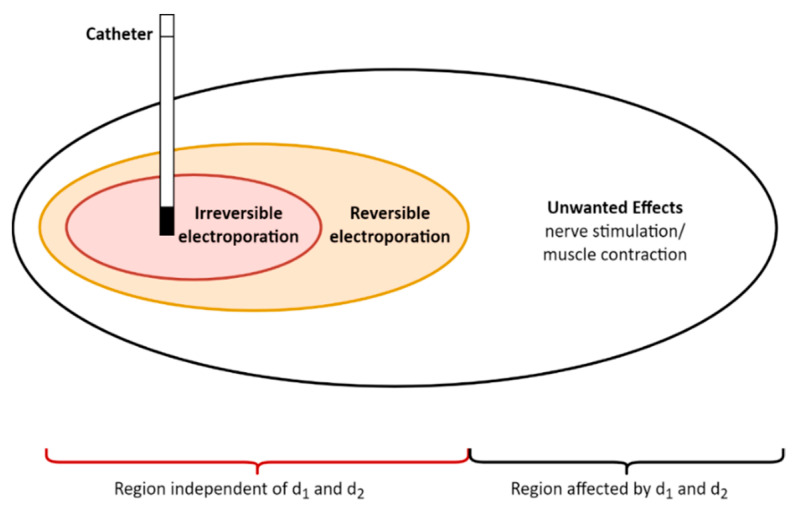
Schematically illustrated regions in and around the treatment site of a patient (regions dependent and independent of the interphase (d_1_) and interpulse delay (d_2_)). The area/volume of nerve excitation extends beyond the volume of cell membrane electroporation due to lower threshold needed for neuromuscular stimulation compared to electroporation thresholds.

In addition to pulse waveforms, one also needs to consider the spatial distributions of electric fields in the context of the different thresholds involved in irreversible electroporation, reversible electroporation and/or nerve–muscle stimulation/activation. While keeping the same pulse parameters, the highest electric field is required for inducing cell death, followed by reversible electroporation, and muscle and nerve stimulation. This also means that the area of ablation by irreversible electroporation will be the smallest, surrounded by reversible electroporation, and affecting/stimulating the nerves passing through the surrounding zone (Figure 10). It is important to note that the spatial distribution of the electric field depends on the biophysical properties of the cardiac tissue, including the inhomogeneity and anisotropy of electric (i.e., conductivity and dielectricity) attributes, as well as on the electrode’s shape, i.e., the catheter design, and whether the pulses are delivered in a unipolar or bipolar fashion [57,58].

During a PFA treatment using specific waveforms, the electrical pulses are delivered to a treatment site using an ablation therapy delivery device composed of a pulse generator and a catheter. Figure 10 schematically illustrates the regions of effect relative to the placement of a catheter delivering treatment in a monopolar fashion. In the red and orange regions (near the catheter), both irreversible electroporation and reversible electroporation of the tissue may occur. In the white region, neuromuscular stimulation and other unwanted effects of the application of PFA pulses can also occur. Irreversible and reversible electroporation occurs in regions independent of the values for d_1_ and d_2_ of the applied pulse protocol (see Figure 8 and Figure 9). However, the occurrence of unwanted effects will be affected by the values for d_1_ and d_2_ of the applied pulse protocol (see Figure 3, Figure 4, Figure 5, Figure 6 and Figure 7).

## 5. Conclusions

Electroporation as an underlying mechanism has emerged as a valuable tool for drug and gene delivery, tumor ablation and, particularly, cardiac ablation (pulsed-field ablation). One needs to consider associated pain and muscle contractions that may be unwanted adverse effects. Such effects may be not acceptable for patients if used for prophylactic treatment, i.e., vaccination. The delivery of high-voltage pulses also has an effect on vascular permeability and blood perfusion as well as autonomic activity. For cardiac ablation procedures, neuromuscular stimulation (e.g., muscle contractions (for example via phrenic nerve stimulation), coughing and induced discomfort) remain as concerns, and may require deeper sedation techniques, which are challenging to implement in some geographics. Potential effects of PFA, including the cough reflex and the effects of the excitation of the nervous system and smooth muscles, remain to be further investigated and understood better. The mechanisms described above shed light on how high-voltage electric pulses used in electroporation treatments, including PFA, may trigger muscle contractions and/or activate pain pathways. With the data obtained from this multifaceted study, we provide guidance/directions in developing new pulse protocols to limit or reduce the adverse effects that occur during PFA, and potentially other applications of electroporation therapies, while the intended therapeutic purposes remain the same. Understanding these processes is crucial for developing strategies to minimize the adverse effects and discomfort associated with electroporation procedures. Continued research and technological advancements in this field will pave the way for more efficient and patient-friendly electroporation treatments in the future, mitigating potential adverse effects.

## Data Availability

The data that support the findings of this study are available within the article.
